# Increased terrigenous input from North America to the northern Mendeleev Ridge (western Arctic Ocean) since the mid-Brunhes Event

**DOI:** 10.1038/s41598-022-19082-y

**Published:** 2022-09-07

**Authors:** Kwangkyu Park, Rujian Wang, Wenshen Xiao, Leonid Polyak, Hyen Goo Cho, Boo-Keun Khim

**Affiliations:** 1 Marine Research Institute, Pusan National University, Busan, 46241 Korea; 2 State Key Laboratory of Marine Geology, Tongji University, Shanghai, 200092 China; 3 Byrd Polar Research Center, The Ohio State University, Columbus, OH 43210 USA; 4 Department of Geology and Research Institute of Natural Science, Gyeongsang National University, Jinju, 52828 Korea; 5 Department of Oceanography, Pusan National University, Busan, 46241 Korea

**Keywords:** Cryospheric science, Palaeoceanography

## Abstract

Mid-Brunhes Event (MBE) occurred at approximately 420 ka between Marine Isotope Stage 11 and 12, and is considered the most pronounced climatic shift during the last ~ 800 kyrs. On the other hand, it is unclear if the MBE was global, despite being observed in the high-latitude Northern Hemispheric cryosphere in terms of climate systems. A 5.35-m long gravity core ARC5-MA01 was obtained from the northern Mendeleev Ridge in the western Arctic Ocean to track the paleoenvironmental changes in terms of the terrigenous sedimentation in response to the glacial-interglacial climate changes across the MBE. Geochemical proxies (biogenic opal, total organic carbon, C/N ratio, carbon isotope of organic matter, and calcium carbonate) of MA01 suggest that the terrigenous input was generally higher during the interglacial periods. Based on a mineralogical examination, most of the terrigenous input was attributed to the abundance of dolomite and the increased kaolinite content from North America. In particular, most paleoceanographic proxies showed that the terrigenous input from North America was enhanced distinctly during the post-MBE interglacial periods. These results suggest that the MBE in the western Arctic Ocean was a global climatic shift closely linked to cryospheric development in North America during the middle Pleistocene.

## Introduction

The glacial-interglacial (G-IG) changes paced by the variations in orbital parameters are the most pronounced climate fluctuations during the Quaternary, which is highlighted by the onset of Northern Hemisphere glaciation^1^. Global terrestrial, marine, and ice archives clearly documented that climatic variations during the recent one third of the Quaternary (~ 800 kyrs) are predominated by 100-kyrs G-IG cycles, particulary during the last four cycles^1,2^. A distinct global climatic shift, called the Mid-Brunhes Event (MBE), occurred at Marine Isotope Stage (MIS) 11/12, which coincides with the largest fluctuation in amplitudes of benthic δ^18^O values over the Quaternary^1,3^. During the interglacial periods, Antarctic ice core records show warmer air temperatures and higher atmospheric CO_2_ concentrations since the MBE, compared with those in the earlier interglacial periods^4,5^. Lang and Wolff^6^ suggested, based on the diverse paleoclimatic data collected from south of ~ 60°N, that the MBE was a global event which is related to the stronger interglacials and terminations during the last ~ 450 kyrs, while the climatic amplitudes during the glacial periods remain less pronounced.

Although climate change during the MBE has been reported worldwide from the terrestrial, marine, and ice core records, only a few studies have been conducted in the Arctic Ocean where the climatic changes fluctuated more severely than the other oceans^7^. For example, based on Mg/Ca ratios in ostracodes from the western Arctic Ocean sediments, Cronin et al.^8^ reported that the thermal maximum events during the transitions from the interglacial to glacial periods since the MBE. They argued that this phenomenon was related to the inflow of warm Atlantic Water, i.e., enhanced Arctic amplification which is the increased warming of Arctic region compared to that of the Northern Hemisphere^7^. Xiao et al.^9^ indicated that preservation of calcareous microfossils improved across the MBE in the western Arctic Ocean, related to a decrease in organic matter export and/or weakened bottom water ventilation along with the development of perennial sea ice. In general, the climatic shift across the interglacials-terminations since the MBE was intensified clearly, but the climate changes during the glacial periods across the MBE are poorly resolved. Such a lack of information is typical for the Arctic Ocean, where calcareous microfossil preservation is poor especially during glacial periods^10^.

Given that the predominent growth of Northern Hemisphere glaciation played an important role in changing the global climate across the Pleistocene^11^, continuous climate records from the Arctic Ocean are a critical subject for unraveling the Arctic glacial history. In particular, the changes in the G-IG amplitudes during the Pleistocene involve various internal forces, including the hypothesis of regolith removal caused by the continental glaciation^11,12^. In this study, we examined geochemical and mineralogical proxies of core ARC5-MA01 (hereafter MA01) in terms of terrigenous sedimentation to reveal the paleoenvironmental changes by orbital-scale glacial activities during the last ~ 840 krys in the northern Mendeleev Ridge of the western Arctic Ocean. Our results reveal an increase of North American terrigenous input to the western Arctic Ocean since the MBE, supporting an global climatic amplification since the MBE.

## Results

**Figure 1:**
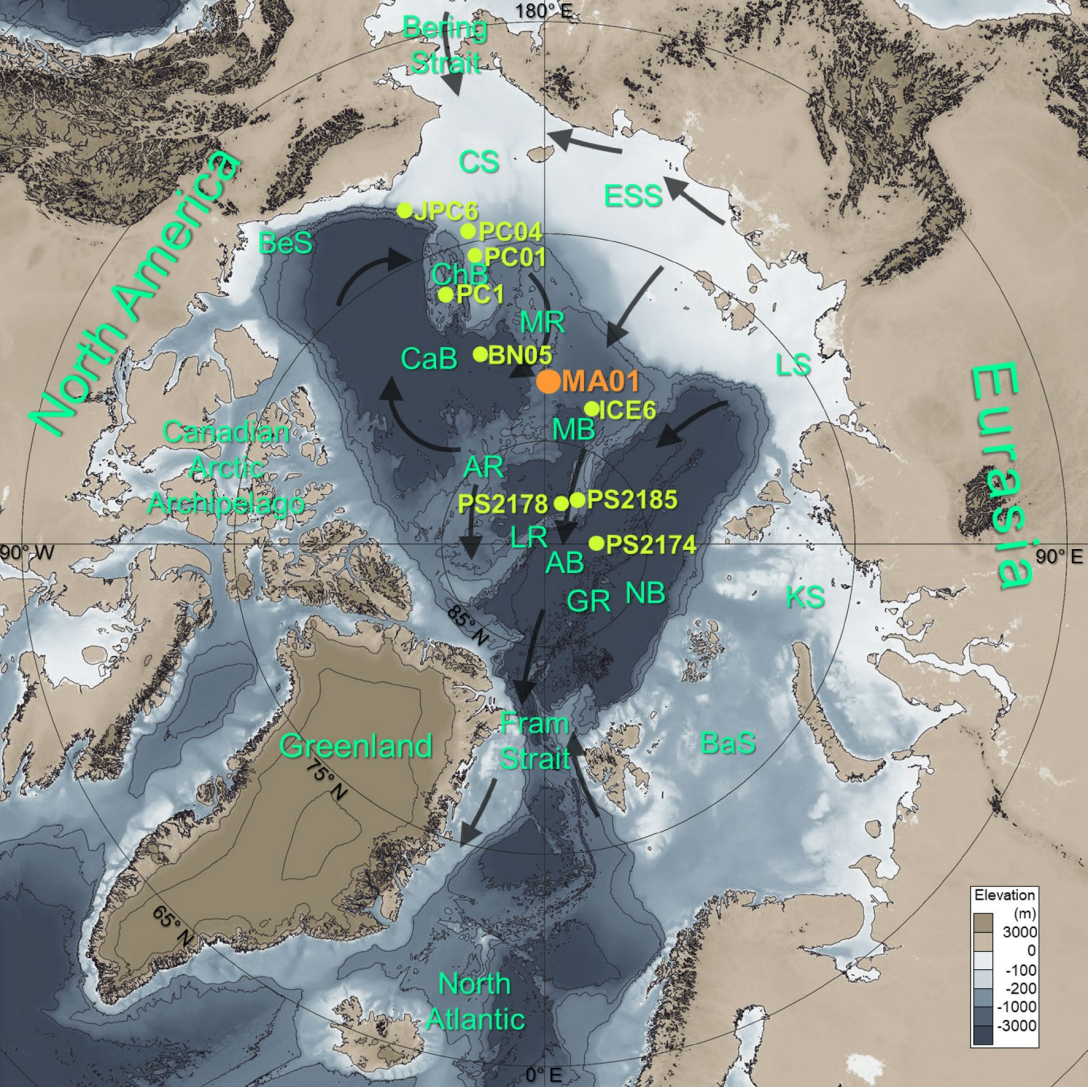
Geographic map showing locations of cores in this study (ARC5-MA01) and referenced sites (Table 1). Black arrows represent the large scale surface circulation system; clockwise Beaufort Gyre in the western Arctic Ocean and Transpolar Drift flowing from the Siberian shelves into the Fram Strait. The bathymetry obtained from the International Bathymetric Chart of the Arctic Ocean (IBCAO V3.1, www.gebco.net) was drew using the open-source geographic software (QGIS, V.3.18.3, www.qgis.org). BeS, Beaufort Sea; CS, Chukchi Sea; ESS, East Siberian Sea; LS, Laptev Sea; KS, Kara Sea; BaS, Barents Sea; ChB, Chukchi Borderland; CaB, Canada Basin; MB, Makarov Basin; AB, Amudsen Basin; NB, Nansen Basin; MR, Mendeleev Ridge; AR, Alpha Ridge; LR, Lomonosov Ridge; GR, Gakkel Ridge.

**Figure 2:**
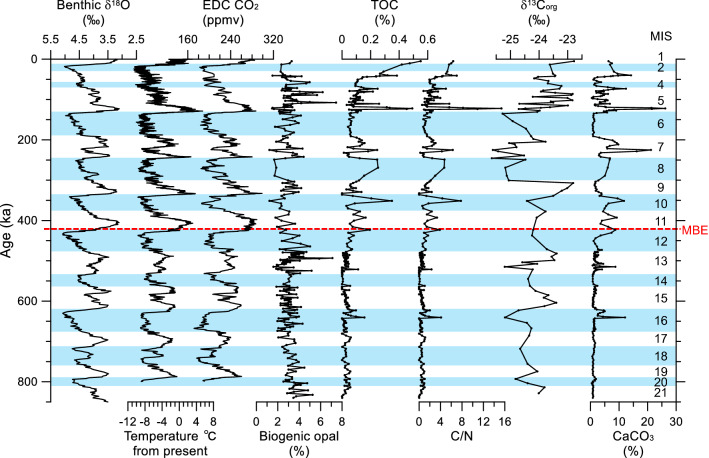
Downcore variation of geochemical results (biogenic opal, TOC, C/N, δ^13^C_org_, and CaCO_3_) of core ARC5-MA01 along with global benthic δ^18^O values^1^ and EPICA Dome C (EDC) records, including air temperature^4^ and atmospheric CO_2_ concentration^5^. Marine isotope stages (MIS) are indicated with blue shadings of glacial periods and white shadings for interglacial periods.

Despite uneven spacing at the temporal intervals, the gechemical results of MA01 show clear G-IG cyclic variations during the last ~ 840 kyrs (Figs. 1 and 2; Table 1). Biogenic opal contents (from 1.2 to 7.4% with an average of 3.1%) are higher during interglacial periods (Fig. 2 and Table 2). Total organic carbon (TOC) contents (from 0 to 0.55%) increased from the glacial periods (*ca*. 0.06%) to the interglacial periods *(ca*. 0.08%) (Fig. 2 and Table 2). In particular, TOC contents are higher and more variable since MIS 11. C/N ratios are higher during the interglacial periods (ca. ~ 1.6) than during the glacial periods (ca. ~ 1.1) and their variations are similar to TOC contents, following higher fluctuations since MIS 11 (Fig. 2). The interglacial δ^13^C_org_ values (− 23.9‰ in average) are slightly higher than the glacial values (− 24.5‰ in average) (Fig. 2 and Table 2). Interestingly, CaCO_3_ content (0.4 to 26.2%) associated with dolomite increases since MIS 16 (Fig. 2). Its average content was higher during the interglacial periods, together with greater fluctuations since MIS 11 (Fig. 2 and Table 2).

**Table 1:** List of cores in this study and reference cited.

Core site	Area	Latitude	Longitude	Water depth (m)	Reference
ARC5-MA01	Mendeleev Ridge	82° 1.86′ N	178° 57.6′ E	2295	This study,^9^
HLY0501-JPC6	Chukchi-Alaskan margin	72° 30.71′ N	157° 2.08′ W	673	^25^
MR09-03 PC04	Chukchi Slope	74° 26.3 N	165° 44.3′ W	370	^14^
MR09-03 PC01	Chukchi Slope	75° 28.1′ N	165° 40.4′ W	558	^14^
MR08-04 PC1	Northwind Ridge	74° 48.50′ N	158° 31.85′ W	998	^17^
ARC4-BN05*	Canada Basin	80° 29.04′ N	161° 27.90′ W	3156	^20^
ARC5-ICE6*	Makarov Basin	83° 37.69′ N	161° 45.84′ E	2901	^22^
PS2178-5	Makarov Basin	88° 1.5′ N	159° 42.2′ E	4008	^13^
PS2185-6*	Lomonosov Ridge	87° 32.2′ N	144° 55.6′ E	1052	^13^
PS2174-5	Amundsen Basin	87° 29.1′ N	91° 32.6′ E	4427	^13^
ARC7-E26*	Mendeleev Ridge	79° 57′ N	179° 41.82 W	1500	^9^
HLY0503-6JPC*	Mendeleev Ridge	78° 17.64 N	176° 59.16 W	800	^9^ and references therein
HLY0503-8JPC*	Mendeleev Ridge	79° 35.58 N	172° 30.12 W	2792	^9^ and references therein
AO96/12–1 PC*	Lomonosov Ridge	87° 5.88 N	144° 46.38 E	1003	^9^ and references therein

The cores marked with asterisk were used in the previous study^9^ for regional stratigraphic correlation.

**Table 2:** Statistical summary of geochemical and mineralogical results of MA01 for interglacial and glacial periods during the last ~ 840 kyrs and post-MBE (after 424 ka) and pre-MBE (before 424 ka).

		B-opal (%)	TOC (%)	C/N	d^13^C_org_ (‰)	CaCO_3_ (%)	PF (#/g/10^3^)	> 154 mm (%)	Kaolinite (%)	Smectite (%)	Illite (%)	Chlorite (%)
Interglacial (MIS 1, 3, …, and 21)	Average	3.2	0.08	1.6	− 23.91	3.6	0.98	2.43	16	7	58	20
	Min	1.2	0.00	0.0	− 25.62	0.4	0.00	0.31	11	1	38	17
	Max	7.4	0.55	15.4	− 22.78	26.2	9.61	17.35	29	14	65	25
	Stdev	1.0	0.10	2.0	0.68	4.0	1.73	2.62	4	2	5	2
Glacial (MIS 2, 4, …, and 20)	Average	3.0	0.06	1.1	− 24.46	2.4	0.10	2.39	14	6	60	20
	Min	1.2	0.00	0.0	− 25.65	0.5	0.00	0.29	11	2	47	15
	Max	5.0	0.35	7.8	− 23.22	12.1	2.14	30.1	23	16	65	23
	Stdev	0.7	0.06	1.3	0.59	2.4	0.39	3.53	2	3	4	2
Post-MBE interglacial (MIS 1, 3, …, and 11)	Average	3.1	0.14	2.7	− 23.88	5.6	1.92	3.74	18	7	56	20
	Min	1.2	0.00	0.0	− 25.62	0.4	0.00	0.47	12	1	38	17
	Max	7.4	0.55	15.4	− 22.78	26.2	9.61	17.35	29	14	64	25
	Stdev	1.0	0.11	2.3	0.74	4.7	2.02	2.88	5	3	6	2
Post-MBE glacial (MIS 2, 4, …, and 10)	Average	2.7	0.10	2.1	− 24.50	3.5	0.30	4.28	16	5	59	21
	Min	1.2	0.03	0.6	− 25.65	0.7	0.00	0.5	13	2	47	19
	Max	4.6	0.35	7.8	− 23.22	11.7	2.14	30.1	23	8	65	23
	Stdev	0.8	0.07	1.6	0.77	3.0	0.65	5.45	3	2	5	1
Pre-MBE interglacial (MIS 13, 15, …, and 21)	Average	3.2	0.03	0.5	− 24.00	1.6	0.00	1.15	13	7	60	20
	Min	1.6	0.00	0.0	− 25.20	0.7	0.00	0.31	11	2	56	17
	Max	7.1	0.19	3.9	− 23.39	8.7	0.01	9.75	17	10	65	24
	Stdev	0.9	0.03	0.5	0.48	1.3	0.00	1.48	1	2	2	1
Pre-MBE glacial (MIS 12, 14, …, and 20)	Average	3.1	0.03	0.7	− 24.42	1.9	0.00	1.46	13	7	60	20
	Min	2.0	0.00	0.0	− 25.19	0.5	0.00	0.29	11	3	51	15
	Max	5.0	0.16	4.1	− 23.73	12.1	0.01	7.02	18	16	64	23
	Stdev	3.2	0.03	1.6	− 23.91	3.6	0.98	2.43	16	7	58	20

**Figure 3:**
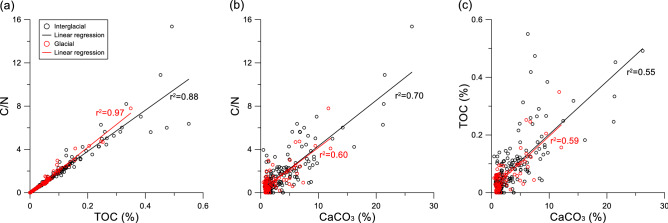
Cross-plots between the geochemical results of core ARC5-MA01. (**a**) C/N vs. TOC, (**b**) C/N vs. CaCO_3_, and (**c**) TOC vs. CaCO_3_ with their correlation coefficient (r^2^) during the glacial (red) and interglacial (black) periods, respectively.

C/N ratio is positively correlated with TOC content in interglacial sediments (r^2^ = 0.88), particularly strong in the glacial sediments (r^2^ = 0.97) (Fig. 3). It is also positively correlated with CaCO_3_ content in both interglacial (r^2^ = 0.70) and glacial (r^2^ = 0.60) periods (Fig. 3). Modest correlation is observed between TOC and CaCO_3_ contents (r^2^ = 0.55–0.59), regardless of the G-IG cycles (Fig. 3). On the other hand, δ^13^C_org_ valus are very poorly correlated with either C/N ratio (r^2^ < 0.01) or TOC content (r^2^ < 0.01). Nonetheless, several low δ^13^C_org_ values (< -25‰) coincide with the peaks of CaCO_3_ content during MIS 16, 13, and 8 to 7 (Fig. 2).

**Figure 4:**
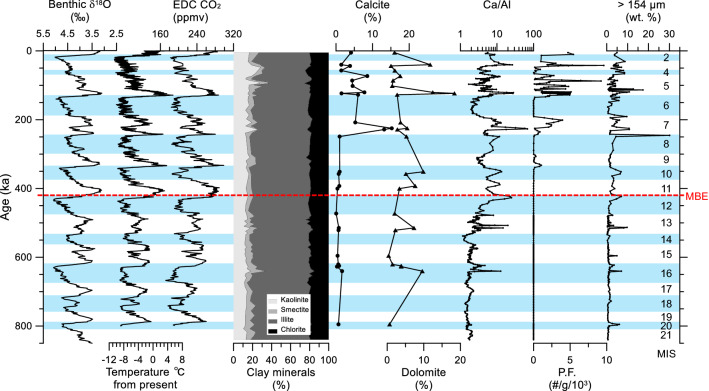
Downcore variation of mineralogical results (four major clay minerals (kaolinite, smectite, illite, and chlorite), calcite and dolomite contents, XRF Ca/Al ratio, planktonic foraminiferal (P.F.) abundance^9^, and coarse-grained (> 154 μm) fraction^9^) of core ARC5-MA01 along with global benthic δ^18^O values^1^ and EPICA Dome C (EDC) records, including air temperature^4^ and atmospheric CO_2_ concentration^5^. Marine isotope stages (MIS) are indicated with blue shadings of glacial periods and white shadings for interglacial periods. Please refer to the supplementary figure S1 for detailed downcore variations of major clay mineral contents.

**Figure 5:**
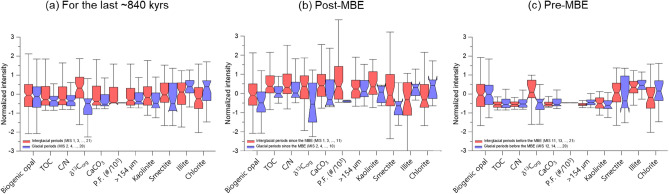
Box–Whisker plots showing the normalized intensity values of geochemical (biogenic opal, TOC, C/N, δ^13^C_org_, and CaCO_3_), micropaleontological (planktonic foraminifera: P.F.), and mineralogical (kaolinite, smectite, illite, and chlorite) data of core ARC5-MA01 for (**a**) the last ~ 840 kyrs, (**b**) the post-MBE, and (**c**) the pre-MBE, respectively. Interquartile ranges multiplied by a factor of 1.5 are indicated as error bars. Red and blue boxes represent the interglacial and glacial periods, respectively.

The relative contents of four major clay minerals show that illite (38–65%, average 59%) is the most abundant component, and other clay minerals (kaolinite, chlorite, and smectite) comprise 15% (11–29%), 20% (15–25%), and 6% (1–16%) in average, respectively (Figs. 4 and S1). Despite low resolution, these clay minerals of MA01 also appear to show G-IG cyclic variations during the last ~ 840 kyrs (Fig. 4). Although variation pattern seems similar among kaolinite, smectite, and chlorite, it is noticed that smectite and kaolinite contents increased during interglacial periods, especially since MIS 11, similar to the patterns of geochemical proxies (Fig. 5). In addition, these variations were differentiated more clearly between the glacial and interglacial periods since the MBE (Fig. 5; Table 2).

Based on the carbonate quantification, most TIC consists of dolomite during the last ~ 840 kyrs (Figs. 2 and 4). The calcite accounts partly for TIC during warm periods since MIS 7, which corresponding to the peaks of planktonic foraminiferal abundance (Fig. 4). In addition, CaCO_3_ peaks associated with dolomite peaks during MIS 16, 13, 7, 5, and 3 coincide with the intervals of high kaolinite content and coarse fraction (Fig. 4).

## Discussions

### Glacial-interglacial changes of geochemical and mineralogical proxies in the northern Mendeleev Ridge during the last 840 kyrs

Geochemical proxies of core MA01 are characterized by G-IG cycles and, particularly, by long-term changes during the last ~ 840 kyrs (Fig. 2). The biogenic opal content, representing the primary productivity of the surface seawater, is fairly low (3.1% in average), which is attributed to the year-round sea ice cover in the central western Arctic Ocean^13,14^. Such low biogenic opal content is similar to those in cores from the central-to-eastern Arctic Ocean (~ 2–4% in average)^13^ and from the Chukchi Rise (3.7–4.4% in average)^15^, which is located northwest and southeast to the Mendeleev Ridge, respectively. In the Northwind Ridge, Polyak et al.^14^, based on foraminiferal assemblages, reported that perennial sea ice condition was established at ~ 0.8 Ma. This is consistent with the overall low primary productivity in this study, indicating that the northern Mendeleev Ridge was covered dominantly with perennial sea ice during the last ~ 840 ka. Despite low, biogenic opal content in this study is slightly different between glacial and interglacial periods, particularly higher (up to ~ 7.4%) during MIS 5, 7, and 13 (Fig. 2). Such difference was also reported from the central Arctic Ocean^13^ and the Chukchi Rise^15^. Schubert and Stein^13^ suggested that the low biogenic opal content during glacial periods was attributed to both reduced marine productivity and increased input of terrigenous sediments. The lower biogenic opal content in the northern Mendeleev Ridge during the glacial periods can be also attributed to the similar reasons of Schubert and Stein^13^. Thus, based on the occurrence of planktonic foraminiferal tests in MA01 which is in line with the G-IG variability of biogenic opal content (Figs. 2 and 4), surface water conditions in the northern Mendeleev Ridge changed between the glacial and interglacial periods during the last ~ 840 kyrs.

TOC content and C/N ratio also represent G-IG changes related to surface water conditions in the northern Mendeleev Ridge (Fig. 2 and Table 2). Similar to the biogenic opal content, TOC content exhibits G-IG variability that is characterized by high content during interglacial periods and low content during glacial periods. The low C/N ratios are mostly due to low TOC content and high proportion of the inorganic nitrogen dominating the total nitrogen^16^. The strong possitive correlation (r^2^ = 0.88 for interglacial periods and r^2^ = 0.97 for glacial periods) between TOC contents and C/N ratios indicates that higher TOC contents were attributed to more contribution of terrestrial organic matters rather than an enhanced surface water productivity in the northern Mendeleev Ridge (Figs. 2 and 3). Such increased contribution of terrestrial organic matter in the northern Mendeleev Ridge is supported by generally low δ^13^C_org_ values (Fig. 2). Previous studies reported that the input of terrestrial organic matter was higher during glacial periods in the western Arctic Ocean^17,18^. In the southern Mendeleev Ridge, Yamamoto and Polyak^17^ suggested that the increased input of terrestrial organic matter during the cold periods was associated with more efficient transport of glaciogenic fine-grained particles from the continent to the western Arctic Ocean. In the Chukchi Borderland, the increase of TOC content was mostly associated with low coarse-grained (> 150 μm) fraction during the cold periods, whereas the marine production of organic carbon more likely increased during the warm periods^15,18^. In contrast, our record from the northern Mendeleev Ridge shows higher TOC content during the interglacial periods rather than the glacial periods (Figs. 2 and 4).

Rella and Uchida^18^ reported that TOC content was negatively correlated with CaCO_3_ and coarse fraction contents in the Chukchi Borderland. For example, high TOC content was accompanied with low CaCO_3_ and coarse fraction contents, resulted from the transport of terrestrial organic matter from CaCO_3_-poor areas such as Siberian margins and/or the less transport of CaCO_3_ to the Chukchi Borderland. In contrast, our geochemical results show that CaCO_3_ content was positively correlated with both TOC content (r^2^ = 0.55–59) and C/N ratio (r^2^ = 0.60–70) (Fig. 3), indicating that both carbonates and organic carbon in the northern Mendeleev Ridge are mainly terrigenous in origin, similar to the study in the eastern-central Arctic Ocean^13,19^. Such terrigenous contribution is evidenced by the peaks of coarse fraction and dolomite contents (Figs. 2 and 4). It indicates that inorganic carbon components consisting mostly of dolomite mainly originated from the Canadian Arctic Archipelago and Mackenzie watershed^9,15,20,21^. Thus, the terrigenous input in MA01 is sourced dominantly from the northern North America. Our finding suggests that the northern Mendeleev Ridge in the western Arctic Ocean is largely affected by sediment deposition from North America. Such increased terrigenous input requires an enhanced Beaufort Gyre in the western Arctic Ocean, leading to more transportation of sediments from North America to the northern Mendeleev Ridge^22^. Thus, the geochemical and bulk mineral results of MA01 indicate that the sediment transport from North America to the western Arctic Ocean, which was stagnated generally during the glacial periods, was activated during the deglacial and stadial periods.

The strong G-IG variabilities are also observed in the variation of major clay mineral compositions of MA01 (Figs. 4 and S1). The overall predominance of illite is well accorded with the result of the previous studies in the western Arctic Ocean^15,21^. Nonetheless, the other minor clay minerals have been used for the provenance studies^15,21,23^. Smectite content in the western Arctic Ocean is very low due to its long distance from the main source areas in the eastern Arctic margins^15,21,24^, although the content is relatively high in the central-western Arctic Ocean^23,25^. Similar to this study in the northern Mendeleev Ridge, very low smectite content was reported from the Chukchi Rise (7–9% in average)^15^ and Canada Basin (3% in average)^21^ in the western Arctic Ocean. Despite low content, the smectite content of MA01 seems different between the glacial and interglacial periods since the MBE (Fig. 4).

In the Chukchi-Alaskan Slope, chlorite was used as a proxy for the Pacific inflow through the Bering Strait^26^. However, chlorite is abundant at the East Siberian margin^16^. Due to the wide distribution of chlorite along the continental margins of the western Arctic Ocean, its G-IG cycles were less detectable in the Chukchi Rise^15^ and Canada Basin^21^ and the northern Mendeleev Ridge of this study area (Fig. 4 and Table 2). In comparison, kaolinite in the western Arctic Ocean reflects a specific source area, i.e., northern Canada and Alaska^27^, and its content was fairly variable among the clay mineral assemblage in the Chukchi Rise and Canada Basin^15,21^. Similarly, the kaolinite content of MA01 shows strong G-IG variability (Figs. 4 and 5; Table 2). Recently, Xiao et al.^23^ argued that the kaolinite was transported from the Franz Joseph Land in the Barents Sea to the Makarov Basin at several glacial/deglacial events including MIS 6 and 4/3. Such increase of kaolinite content was observed at MIS 4/3 but not at MIS 6 in MA01 (Fig. 4), presumably due to the different sedimentation regimes between the northern Mendeleev Ridge and the Makarov Basin. It should be noted that distinct kaolinite peaks of MA01 coincided with the detrital carbonate peaks (e.g., calcite, dolomite, and XRF-Ca/Al) indicative of the enhanced terrigenous input from North America during MIS 16, 13, 7, and 5 (Figs. 2 and 4). All these imply that the clay-sized particles were also delivered together with the coarse fraction from North America to the northern Mendeleev Ridge.

Park et al.^15^ suggested that high kaolinite content in the Chukchi Rise was attributed to more terrigenous input from North America. The terrigenous input was more active during the deglacial period rather than the peak glacial period when the terrestrial glaciation and sea ice were most intensive^15,28^, which strongly reduced sediment transportation to the western Arctic Ocean^29,30^. Accordingly, in the northern Mendeleev Ridge, terrigenous input increased during the deglacials and/or interglacial periods when more terrigenous sediments could be transported efficiently from North America via iceberg and/or sea ice under open water condition.

### Increased terrigenous input to the northern Medeleeve Ridge since the MBE and its paleoenvironmental implications

The geochemical and mineralogical results of MA01 demonstrate an amplification of G-IG contrast across the MBE (Fig. 5 and Table 2). Particularly, TOC and CaCO_3_ contents, C/N ratio, and kaolinite content associated with dolomite peaks were distinctly high during the post-MBE interglacial periods, suggesting enhanced terrigenous inputs from North America (Figs. 2, 4, 5 and Table 2). According to terrestrial and marine records as well as modeling results, continental ice sheets in North America have grown remarkably during the late Quaternary^31–34^. In the northern North Atlantic, glaciogenic marine sediments were observed since the Pliocene–Pleistocene transition, while the detrital carbonate sediments from North America started to appear clear since MIS 16^35^. Similarly, in the western Arctic Ocean, detrital carbonate occurred distinctly since MIS 16, indicating more intensive expansion of North American ice sheets^9,36^. Hence, the sediment input originated from North America into the western Arctic Ocean increased during the middle Pleistocene as a result of the development of the North American ice sheets. Such increased terrigenous input to the northern Mendeleev Ridge is evidenced by our multiproxy results that are characterized by more distinct G-IG fluctuation since the MBE (Fig. 5 and Table 2), while the first extensive development of North American ice sheets occurred at MIS 16 (Fig. 4).

Many records regarding the climatic shift during the post-MBE (since MIS 11) are distinguished by strong interglacial conditions represented by warmer seawater temperatures, higher atmospheric CO_2_ concentrations, and less ice volume^1,4–6,37^ (Figs. 2 and 4). Such climate shift was also accompanied with strong glacial conditions including colder atmospheric temperature and lower CO_2_ concentration^4,5^ (Figs. 2, 4). A possible explanation to the MBE is related to the gradual removal of continental regoliths in the Northern Hemisphere during the Quaternary^11,12^. Regolith removals, resulting from the repeated glaciations, facilitated silicate weathering of the freshly exposed bedrocks and, thus, accelerated the removal of atmospheric CO_2_ concentration, which subsequently enhanced global cooling by positive feedback^11,12^. Clark et al.^11^ demonstrated that the long-term climate change superimposed G-IG cycles during the Quaternary is closely linked to the increase in ice volume since the early Pleistocene. This successive climatic evolution eventually occurred globally as the change of G-IG cycles from ~ 41 to ~ 100 kyrs that completed during the middle Pleistocene (~ 700 ka)^11^. Based on the geochemical and mineralogical proxies of MA01, such climatic changes related to ice sheet development on North America occurred first in the northern Mendeleev Ridge at MIS 16 (~ 640 ka) and fluctuated distinctly since the MBE (Figs. 2, 4, 5). Hence, because the terrestrial-origin sediment deposition increased since the MBE as a result of the development of the North American ice sheets adjacent to the western Arctic Ocean, our results support the regolith removal hypothesis^11,12^. Then, the remaining question is why more terrigenous sediments were deposited during interglacial periods in the northern Mendeleev Ridge of the western Arctic Ocean.

The cooling in the northern Mendeleev Ridge across the MBE can be found not only by the increase of terrigenous input but also by the change of the surface water condition. Despite the cause of the changes in calcareous microfossil abundance in the central-to-western Arctic Ocean remains unclear^38,39^, distinct G-IG contrast of surface water conditions are inferred by biogenic opal content and planktonic foraminiferal abundance of MA01 since MBE (Figs. 2, 4, 5). The G-IG contrast was particularly amplified since MIS 11 with a decrease (2.7% in average) of biogenic opal content during the glacial periods (Fig. 5; Table 2), which indicates post-MBE colder glacial conditions. Such productivity difference seems to be related to the change in seawater temperature and sea ice cover between the glacial and interglacial periods. Cronin et al.^40^ suggested that perennial sea ice started to develop during the interglacial period across the MBE based on calcareous microfossil assemblages that represented the faunal transition in the western Arctic Ocean. Later, thick perennial sea ice conditions were settled during the glacial periods^40^, corroborating a gradual cooling regime across the MBE in the northern Mendeleev Ridge. Based on the changes of microfossil abundance in the central-western Arctic Ocean, Marzen et al.^39^ reported that sea-ice related productivity increased during the interstadial periods since the MBE, with modulation by ~ 100 kyrs of G-IG cycles. It suggests that the climate variability was more sensitive in the central-western Arctic Ocean during the strong interstadials compared to the global climate variablity^39^, as an example of Arctic amplification at geological time-scale^7^. Due to this high sensitivity to climate change, not only 100 kyrs cycle of G-IG contrast but also shorter (< 20 kyrs) stadial/interstadial variations are recorded in the northern Mendeleev Ridge, leading to more terrigenous input during the interglacial periods. Such high sensitivity to climate changes from global cooling eventually occurred as distinct G-IG contrast in the northern Mendeleev Ridge since the MBE. Continuous regolith removal during the Quaternary has been accelerated since the middle Pleistocene^37^. Such condition continued to supply more terrestrial sediments to the northern Mendeleev Ridge in the western Arctic Ocean during warmer and longer post-MBE interglacial periods and multiple stadial/interstadial transitions.

## Conclusions

In this study, diverse geochemical and mineralogical proxies of MA01 were analyzed to track paleoenvironmental changes in terms of terrigenous sediment deposition related to the glacial history during G-IG climatic changes for the last ~ 840 kyrs. The overall features of geochemical and mineralogical results in the northern Mendeleev Ridge accorded with the previous results reported in other regions of the western Arctic Ocean. But partial difference from the central-western Arctic Ocean seems attributable to the regional difference in depositional environment. Our results highlighted that the most significant difference between the glacial and interglacial periods was the terrigenous input from North America and sea surface conditions during the post-MBE. This finding is well aligned with the regolith removal hypothesis, which has been proposed as one of the primary causes of global cooling and completion of ~ 100 G-IG world during the Pleistocene. We suggest that the post-MBE G-IG contrast in the northern Mendeleev Ridge resulted from global cooling and internal positive feedback, both of which are closely related to the cryospheric development in North America. Although the strong G-IG contrast during the post-MBE seems observable in the northern Mendeleev Ridge, diverse regional effects should be considered using further paleoceanographic proxies for better understanding the paleoenvironmental history in the western Arctic Ocean.

## Materials and methods

A 5.35 m-long gravity core MA01 was taken from the northern Mendeleev Ridge (178° 58′ E 82° 02′ N, 2295 m deep) by R/V Xue Long during scientific cruise CHINARE-V in 2012 (Fig. 1). Core sediments were subsampled at 2 cm intervals at Tongji University (China) and ground after freeze-drying at Pusan National University (Korea) to proceed the laboratory analyses. Xiao et al.^9^ reported color reflectance, bulk elemental composition, coarse fraction (> 150 μm), foraminiferal abundance, AMS ^14^C dating, and paleomagnetic inclination of MA01.

### (Chrono-) stratigraphy

The uppermost intervals (0–23 cm) of core MA01 were constrained by AMS ^14^C dates to MIS 1–3. For the older sediments, the stratigraphy was determined by lithostratigraphic and biostratigraphic regional correlations and tunning of Mn content to global climate variability^9^. The core stratigraphy was correlated to the earlier published records from a wide area of central-western Arctic Ocean, including the Mendeleev and Lomonosov Ridges and the Makarov and Canada Basins. The lowermost biostratigraphic marker constrains MIS 11^9^. Beyond MIS 11, the stratigraphy largely relies on the Mn-based tunning due to the lack of identifiable stratigraphic marker. Thus, the stratigraphy below MIS 11 interval seems tentative. Nonetheless, several lithostratigraphic features comfirm that the stratigraphy of MA01 is plausibly acceptable, such as the first detrital carbonate occurrence corresponding to MIS 16^35^ and the distinct cyclic Mn variabilities, general lithologic feature of middle-to-late Pleistocene sediments in the Arctic Ocean^14,36^. In this study, we adopted the same age model of Xiao et al.^9^ to interpret our geochemical and mineralogical data of MA01.

### Geochemical measurements

Geochemical properties were analyzed on a total of 271 samples. Biogenic silica (Si_BIO_) content was measured using the molybdate blue spectrophotometer following the wet-alkaline sequential extraction method modified from DeMaster^41^. The biogenic opal content was calculated by multiplying Si_BIO_ by 2.4^42^. The analytical error of biogenic opal content is less than ± 1%.

Total inorganic carbon (TIC) content was measured using UIC CO_2_ Coulometer CM5014. The analytical error of TIC is ± 0.1% as relative standard deviation. CaCO_3_ content was calculated by multiplying TIC contents by 8.333 (the ratio of C to CaCO_3_). Total carbon (TC) and total nitrogen (TN) contents were measured using an elemental analyzer Flash 2000. The analytical errors are less than ± 0.1% and ± 0.01%, respectively. TOC content was calculated as the difference between TC and TIC and C/N ratio was calculated by TOC/TN.

Carbon isotope of sediment organic matter (δ^13^C_org_) was measured from 82 horizons using Europa Scientific 20–20 Elemental Analyser-Isotopes Ratio Mass Spectrometer (IRMS) at Iso-Analytical Ltd (UK). Expressed conventional delta notation is per mil deviation from the Vienna Pee Dee Belemnite (V-PDB) for carbon isotope. Precision for carbon isotope is ± 0.1‰.

### Clay mineral analysis

Major clay mineral compositions (smectite (17 Å), illite (10 Å), kaolinite, and chlorite (7.1 Å)) of fine-grained sediments were measured for 90 samples. Organic matters of bulk sediments were removed by 6% hydrogen peroxide solution. After wet-sieving through a 63 μm mesh, clay particles (< 2 μm) were extracted by settling technique based on Stoke's Law. The extracted clays were applied on glass slide into orientation and air-dried^43^. The dried slides were scanned using X-ray diffractometer (XRD; SIEMENS/BRUKER D5005) at Gyeongsang National University (Korea) and repeated after ethylene glycol-saturating at 60 °C during 24 h^44^. Relative contents of four major clay minerals were calculated semi-quantitatively following the scheme of Biscaye^45^. The proportion of kaolinite and chlorite was determined by their peak areas (3.58 Å and 3.54 Å), respectively^46^.

### Quantitative analysis for carbonates

Contents of carbonate minerals (calcite and dolomite) were quantified for the selected 14 samples that were decided by the prominent peaks of coarse fraction and TIC content. Bulk sediment powders were analyzed using the same XRD. Following the assumption that the carbonate minerals of the Arctic Ocean sediments consist mostly of calcite and dolomite^34^, TIC content was simply divided into calcite and dolomite proportions as below.

$$\text{calcite}\,({\%})=\frac{{\text{RI}}_{\text{calcite}}}{({\text{RI}}_{\text{calcite}} + {\text{RI}}_{\text{dolomite}})}\times \text{TIC} \times 8.333$$

$$\text{dolomite}\,({\%})=\frac{{\text{RI}}_{\text{calcite}}}{({\text{RI}}_{\text{calcite}} + {\text{RI}}_{\text{dolomite}})}\times \text{TIC}\times 7.67$$

where RI represents relative intensity of calcite (3.04 Å) and dolomite (2.89 Å).

## Supplementary Information

Supplementary Information 1.

Supplementary Information 2.

## Data Availability

All data generated or analysed during this study are included in this published article and its supplementary file (MA01_dataset_202201_SR.xlsx).
