# Supplementary material for: Increased terrigenous input from North America to the northern Mendeleev Ridge (western Arctic Ocean) since the mid-Brunhes Event
Source: Sci Rep. 2022 Sep 7;12:15189. doi: 10.1038/s41598-022-19082-y (PMC9452527; doi:10.1038/s41598-022-19082-y)
Supplement: Supplementary file 2 — Supplementary Information 2. [file 41598_2022_19082_MOESM2_ESM.pdf]

Supplemental Material

Increased terrigenous input from North America to the northern Mendeleev Ridge (western Arctic Ocean) since the mid-Brunhes Event

Kwangkyu Park[1], Rujian Wang[2], Wenshen Xiao[2], Leonid Polyak[3], Hyen Goo Cho[4], Boo-Keun Khim[5,*]

[1]Marine Research Institute, Pusan National University, Busan, 46241, Korea

[2]State Key Laboratory of Marine Geology, Tongji University, Shanghai, 200092, China

[3]Byrd Polar Research Center, The Ohio State University, Columbus, OH 43210, USA

[4]Department of Geology and Research Institute of Natural Science, Gyeongsang National University, Jinju, 52828, Korea

[5]Department of Oceanography, Pusan National University, Busan, 46241, Korea

*Corresponding author (bkkhim@pusan.ac.kr)

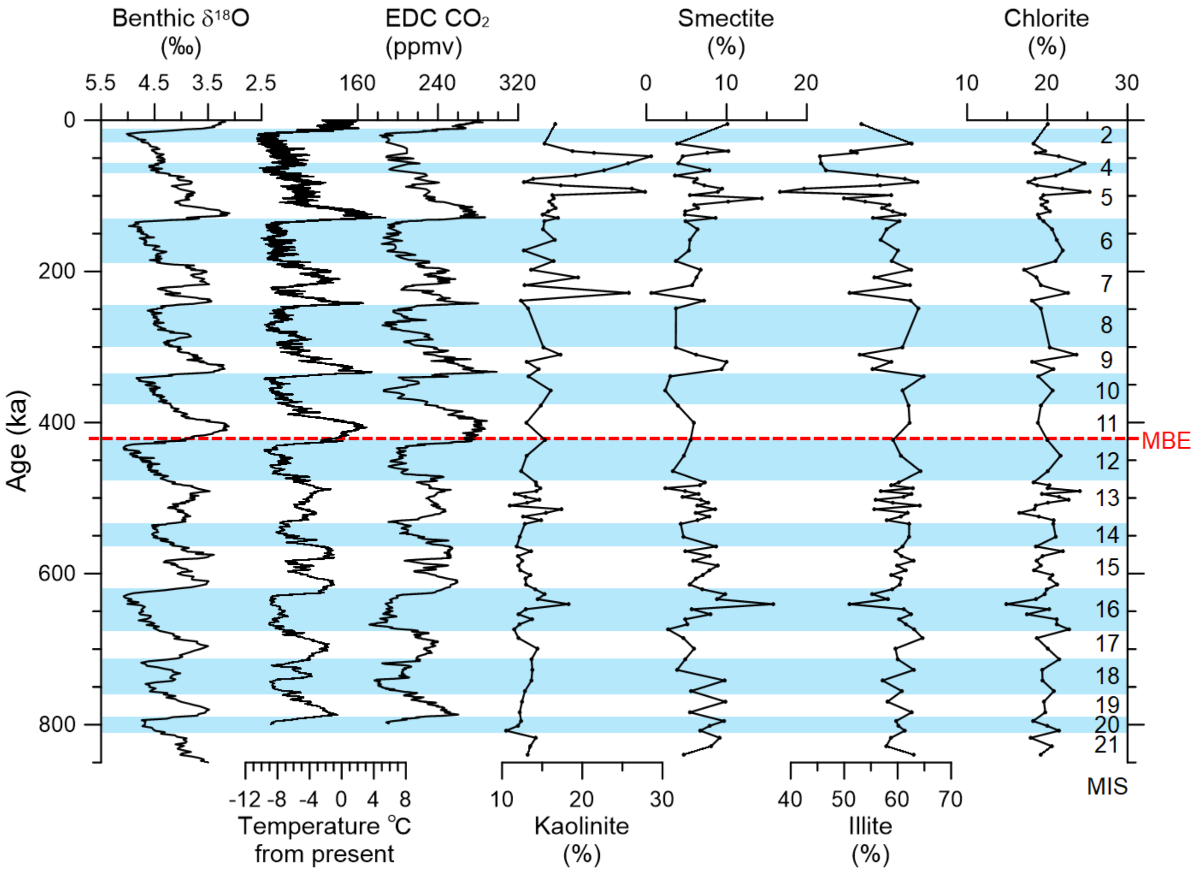

Figure S1. Downcore variation of the contents of four major clay minerals (kaolinite, smectite, illite, and chlorite) of core ARC5-MA01 along with global benthic $\delta^{18}O$ values[1] and EPICA Dome C (EDC) records, including air temperature[4] and atmospheric CO_2 concentration[5]. Marine isotope stages (MIS) are indicated with blue shadings of glacial periods and white shadings for interglacial periods.
